# Haptic sound-symbolism in young Spanish-speaking children

**DOI:** 10.1371/journal.pone.0220618

**Published:** 2019-08-08

**Authors:** Alberto Falcón, Ulianov Montano, Mariel Tavira, Osmara Domínguez-Gallegos

**Affiliations:** Laboratorio de Comunicación Humana y Cognición, Facultad de Comunicación Humana, Universidad Autónoma del Estado de Morelos, Cuernavaca, Morelos, México; The University of Electro-Communications, JAPAN

## Abstract

Haptic sound symbolism has been found in adults, between ideophones and various textures, between words and shape, and between written words and texture. However, associations between the *sound* of nonwords and features other than shape and in early stages of development have been less explored. The present study investigates the haptic manifestation of sound symbolism in an early developmental stage. We examined associations between nonwords and the rough-smooth tactile dimension in 3.5-to-4.5-year-old children. Two experiments were conducted: a pointing selection task and a naming task. Sound symbolic associations were found in the naming task, but not in the pointing task. On the other hand an unexpected bias towards smoothness was found in the pointing task. We explain these results by suggesting that the articulation of nonwords may facilitate or intensify sound symbolism, and that hedonic biases are manifested in the pointing task.

## Introduction

Sound symbolism is the systematic association between the sound of a word and features such as the form or size of the object to which the word refers. There is abundant evidence that confirms the existence of the phenomenon. The *sound symbolism* phenomenon was first demonstrated by Sapir [1] and Kohler [2]. Kohler’s results were later replicated utilizing the nonwords *bouba* and *kiki*. Hence, recent studies refer to sound symbolism as the *bouba-kiki effect* [3,4]. The importance of sound symbolism is testified by the fact that some authors argue that it is a universal phenomenon [5,6], and that it plays a role in language acquisition. Diverse approaches also suggest that the phenomenon may be based on biases in the psychological [7], neurological [8] or biological [9,10] constitution of human beings.

Linguistic studies show that in addition to the well-known phonetic symbolism (association between meaning and phonemes) there are symbolic associations with intonation, prosody and grammar [11–13]. Psycholinguistic evidence shows that sound symbolism is relevant even to language learning [14–16]. It is also present cross-culturally; for instance in Tanzanian children [17]; and in Himba participants (in Namibia), who have no written language, ruling out possible orthographic effects [3].

The present study investigates the tactile manifestation of sound symbolism in an early stage of development. Regarding the developmental aspect, sound symbolism seems has been found in early stages of development, which is relevant since this suggests that the phenomenon may be universal and biologically based. Maurer et al. [18] found sound symbolic associations in 2.5-year-old children. Asano et al. [8] present neurophysiological evidence that 11-month-old infants integrate visual and spoken-word inputs. Ozturk et al. [19], using a preferential looking paradigm that presented shapes paired with nonwords, found sound-shape associations in 4-month-old infants. Similar results are reported by Peña [20]. Sound symbolism also facilitates word learning in toddlers [21,22]. Tzeng et al. [23] investigated the development of sound symbolism. They found that 3-year-olds exhibited chance performance in the usual round/spiky task, whereas 5- and 7-year-olds exhibited sound symbolic effects; suggesting that sound symbolism develops with experience. Although sound symbolism has been found in infants and toddlers, there are also results that show that the effect is sensitive to factors such as task difficulty. For instance, Fort et al. [24], using a preferential looking paradigm in which they presented two shapes and one nonword, failed to find any significant effect in 4-month-old infants. Results were attributed to the difficulty of the task.

Now, sound symbolism is not limited to sound and shape. Research on systematic crossmodal associations have found evidence of associations between nonwords and flavors [25–28], shapes and odors [29,30], odors and musical notes [25,31], and pitch and diverse visual features [26,32,33]. Interestingly enough, there is little research on the associations between tactile and other sensory features. Sound symbolism was first demonstrated by studying sound-size and sound-shape associations [1,2]. Size and shape are features that are accessible through the visual and tactile modalities: one can see as well as touch the round or spiky shape of an object. It thus stands to reason that the tactile modality should be among the first lines of inquiry on extra-visual sound symbolism. This contrasts with the scarcity of studies on tactile sound symbolism. Among the cross-modal studies relevant to tactile sound symbolism we can cite the following: there are associations between visual lightness and vibro-tactile frequency [34], between smoothness and softness, and color luminance and chroma [35]. High-pitched sounds are rated as sharper, rougher, harder, colder, drier and lighter than low-pitched sounds [36].

In addition to tactile cross-modal correspondences, studies on proper tactile sound-symbolism (i.e., on the association of *words*, including nonwords, ideophones and words denoting psychological states, and tactile dimensions) are also very few. Gick and Derrick [37] found that tactile and auditory information is integrated in speech perception. They applied controlled air puffs on their participants’ skin while the participants simultaneously heard spoken syllables. Tactile stimulation modulated speech perception; for instance, participants misheard *b* as *p* under stimulation.

Sakamoto and Watanabe [38] found tactile associations in Japanese ideophones. Using 120 different materials as tactile stimuli, participants were asked to express their sensations using Japanese ideophones in relation with dimensions such as comfort/discomfort, bumpy/flat, rough/smooth, hard/soft, non-elastic/elastic, slippery/sticky, dry/moist, and warm/cold. It was found that positive ratings tended to correspond to /u/, and negative ratings to /i/ and /e/. Also, voiced consonants (e.g., /dz/ and /g/) corresponded to roughness, and voiceless consonants (e.g., /ţ/, and /s/) to smoothness; among other similar associations.

Sound symbolism with nonwords rather than ideophones, has been reported by Fryer et al. [39]. Participants were asked to touch paper and 3D models of the standard spiky or rounded shapes utilized in *bouba-kiki* studies. When asked to name each model either as *kiki* or *bouba*, the rounded shapes were systematically associated with *bouba*, and the spiky shapes with *kiki*. Etzi et al. [40] investigated associations with textures of everyday materials (cotton, satin, tinfoil, sandpaper and abrasive sponge). Participants were stroked with samples of the materials and asked to rate them according to rating scales anchored in word pairs, for instance, *bouba/kiki* or *light*/*heavy*. Sound symbolic associations were observed, especially between rough textures and *kiki*-type words. It must be pointed out that Etzi et al.’s study examined associations utilizing only *written* words. The study is thus mute about *auditory*-tactile associations. Regarding auditory-tactile associations, Domínguez-Gallegos [41] explored the hypothesis that there may exist an analogy between the friction in the mouth involved in producing fricative consonants (such as /f/, /Ө/, /s/, /j/, /x/, /ð/) and the friction experienced in touching rough or smooth materials. Domínguez-Gallegos found evidence that fricative-rich nonwords are associated with tactile rough perceptions, whereas fricative-free nonwords are associated with smooth perceptions.

To the best of our knowledge, the studies of Fryer et al. [39], Etzi et al. [40], and Domínguez-Gallegos [41] are the only studies reporting evidence for haptic sound symbolism with nonwords ([38,42] study ideophones). The present study explores an auditory-tactile association not previously addressed, and it does so in young children, in an early developmental stage.

### Present study

In the present study we test for auditory-tactile associations in young children. Since Fryer et al. have studied sound-shape associations, and Etzi et al. *written* nonword-texture associations; we investigate associations between sound (in the *auditory* modality) and texture. We explore two aspects of tactile sound symbolism. First, we examine the phenomenon in 3.5-to-4.5-year-old children. Second, since sound symbolic effects have been shown to be sensitive to task difficulty, we implemented an experimental design with two different types of cognitive demands. In the first experiment, henceforth *Experiment 1*, children were asked to choose which texture corresponded to a given nonword (the children responded by pointing to the texture, hence we refer to this task as a *pointing task*). In the second experiment, *Experiment 2*, children were asked to assign one of two nonwords to the texture indicated by the experimenter (we refer to this as a *naming task*).

In principle, the naming task is more demanding than the pointing task. The naming task involves not only making a decision that taps into the cross-modal associations, but it also involves maintaining the nonwords in mind (which are unfamiliar words and thus difficult to remember), and pronouncing the words correctly. This combination of decision making, information maintenance, and articulation is more resource-demanding than the pointing task. Therefore, it is possible that in the naming task the sound symbolic effect may manifest itself in an attenuated manner.

Thus, based on previous studies, specifically Domínguez-Gallegos [41], we predict that fricative-rich nonwords will be associated with the rough stimulus, and fricative-free nonwords with the smooth stimulus.

## Method

The method was adapted from Maurer et al. [18]. In their study, Maurer et al. explored sound symbolism in 2.5-year-old children using pairs of rounded and pointed shapes and four pairs of nonwords differing in vowel content. For our purposes, the most relevant aspect of Maurer et al.’s study is the way they adapted the forced choice task in order to make it accessible to young children They introduced stuffed toys to play with the children. Maurer et al.’s study consisted of eight forced-choice trials, four experimental trials and four validity check trials. In the validity check trials the experimenter asked the children to help him find his friends by showing the children pictures of the target toys (green or yellow rabbits). In the experimental trial, to test the sound symbolic associations, the children were asked to pick rounded or pointed figures corresponding to his friend’s “funny name” (one of their eight nonwords).

Inspired by Maurer et al.’s validated procedure; we used toys and play to engage children in the different phases of the experiments, as described below. However, there are three significant differences in our procedure. First, toys, story, stimuli and materials were designed to test for the rough-smooth dimension in the tactile modality. Second, the method was adapted for Spanish-speaking children, utilizing nonwords that were previously validated with adults. Third, our method involved two experiments; a pointing task in Experiment 1, and a naming task in Experiment 2.

### Experiment 1. Pointing task

#### Participants

Twenty-nine healthy children whose mean age was 3 years and 11 months (SD = 3 months and 17 days; range = 3.5–4.5 years) participated in Experiment 1. Participants were recruited from two public schools: a rural school in the State of Morelos, Mexico, and an urban school situated in the metropolitan area of Cuernavaca City, also in Morelos. Children were excluded if they had any type of learning or neurological disability or if they were speakers of a language other than Spanish. All children in the age range were recruited for participation after previous request to the group caretaker. Informed consent from parents and school authorities on behalf of children were obtained. The protocol was approved by the Research Ethics Committee of the Faculty of Human Communication of the UAEM. Experiment 1 was conducted on May 2017.

Schools belonged to neighborhoods with a low to medium socio-economic status. Although these schools were located within a relatively small region of México, their linguistic and other relevant characteristics do not exhibit any significant bias.

#### Stimuli and materials

In both experiments, the pairs of contrasting nonwords */krexis/* and */nunum/* (note that the phoneme */x/* is a fricative phoneme; it is the Spanish sound corresponding to the letter *j*, *Jota*, as in Spanish *joven*), and */xikres/* and */munmu/* were tested for possible tactile associations in a counter-balanced manner. The nonwords do not resemble the Spanish words for rough (/rasposo/, /rugoso/, /aspero/) and smooth (/liso/, /suabe/, /terso/). However, it must be pointed out that some phonemes, for instance /r/ or /s/, are shared by the Spanish words and the test nonwords. Now, this does not represent a bias that may influence the test, since the phonemes are shared by the words for rough (note that /r/ and /s/ appear in /rasposo/), as well as by the words for smooth (/r/ and /s/ appear in /terso/). In addition words had been previously validated with adult Spanish speakers by Domínguez-Gallegos [41].

Domínguez-Gallegos explored the hypothesis that there may be an analogy between the friction action in pronouncing fricative consonants such as */f/*, */Ө/*, */s/*, */j/*, */x/*, */ð/* and the friction experienced in touching the materials. Utilizing the Random Word Generator software, Domínguez-Gallegos generated 500 nonwords that were either rich in fricatives or with no fricatives. Eighteen nonwords were selected and tested for tactile associations with Spanish speaker adults. */krexis/* and */nunum/* showed the most robust associations with roughness and smoothness, respectively.

Tactile stimuli for validation trials consisted of 4 pairs of plastic animal toys (2 tigers, 2 elephants, 2 lions and 2 zebras). In each pair, one toy could be distinguished from the other only by means of touching their *smooth* or *rough* feet (the feet’s smooth or rough textures were the same as those in the testing stimuli).

**Fig 1:**
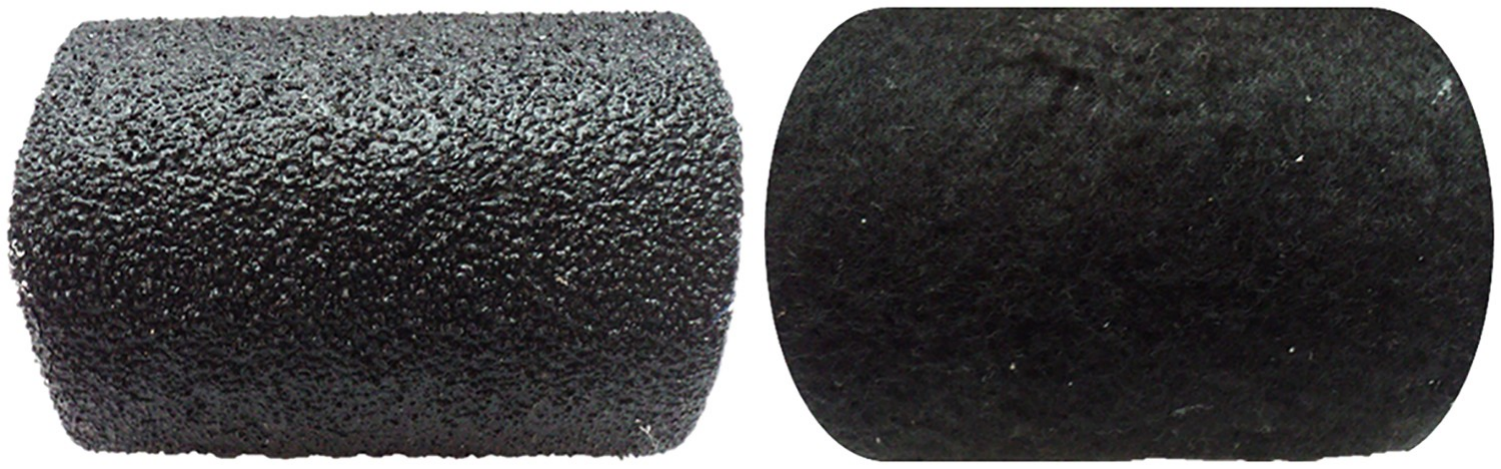
Tactile stimuli for test trials. Coarse grain sandpaper (left) and polar fleece fabric (right). These textures were not visible to the children, inside cardboard cylinders.

**Fig 2:**
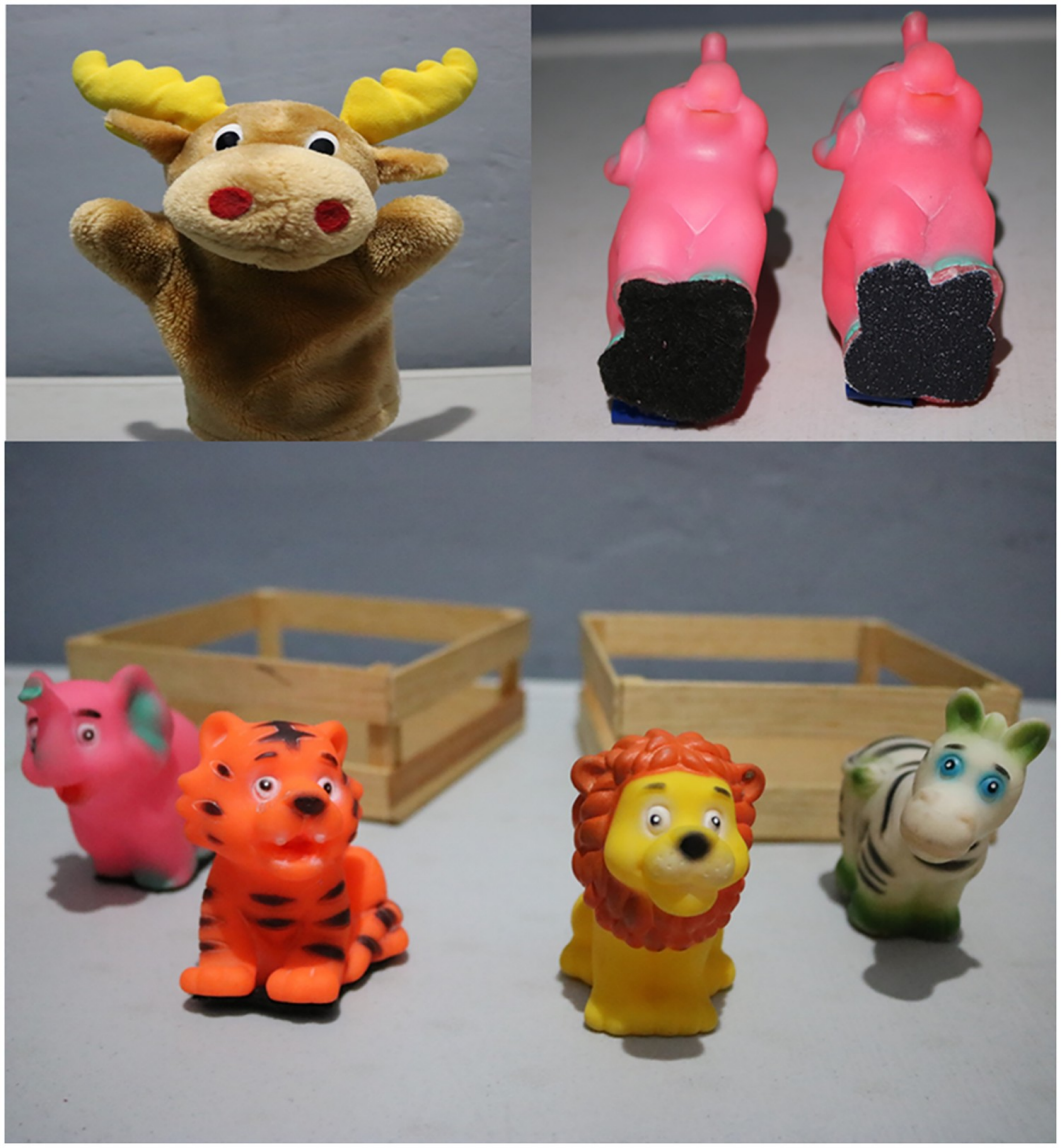
Materials for test trials. Cylinders containing rough and smooth stimuli, designed so that the stimuli are not visible to the child. Each cylinder encloses one type of stimulus, counter-balanced distributed to the left or right.

For test trials, the rough stimulus was provided by aluminum oxide sandpaper (grit 40), which was the coarsest available (Fig 1, left), chosen as in [43]. The smooth stimulus was provided by polar-fleece fabric (Fig 1, right). Each material was applied on a cardboard roll, as shown in Fig 1, left and right. To provide tactile but not visual stimulation, we devised two colored cylinders with small openings so that children could not see inside the cylinder, but they could reach inside and touch the materials with two fingers (see Fig 2).

#### Procedure

The experimenter visited schools during regular school days. Children were tested one by one in an adjacent room. Before starting with the validation and test trials, children played with the toys and the experimenter for 5 minutes.

Experiments consisted of a total of 4 validation trials and 4 test trials. Trials were presented in the following order in both experiments: validation trial 1 (VT1), test trial 1 (TT1), validation trial 2 (VT2), test trial 2 (TT2), validation trials 3 and 4 (VT3 and VT4) and test trials 3 and 4 (TT3 and TT4). To be included in the final analysis, participants should have correctly answered all of the four validation trials.

The aim of the validation trials was to familiarize children with the textures, to explore their vocabulary and linguistic competences, to make sure that they were motivated to cooperate with the experimenter, and that they were able to distinguish between textures.

**Fig 3:**
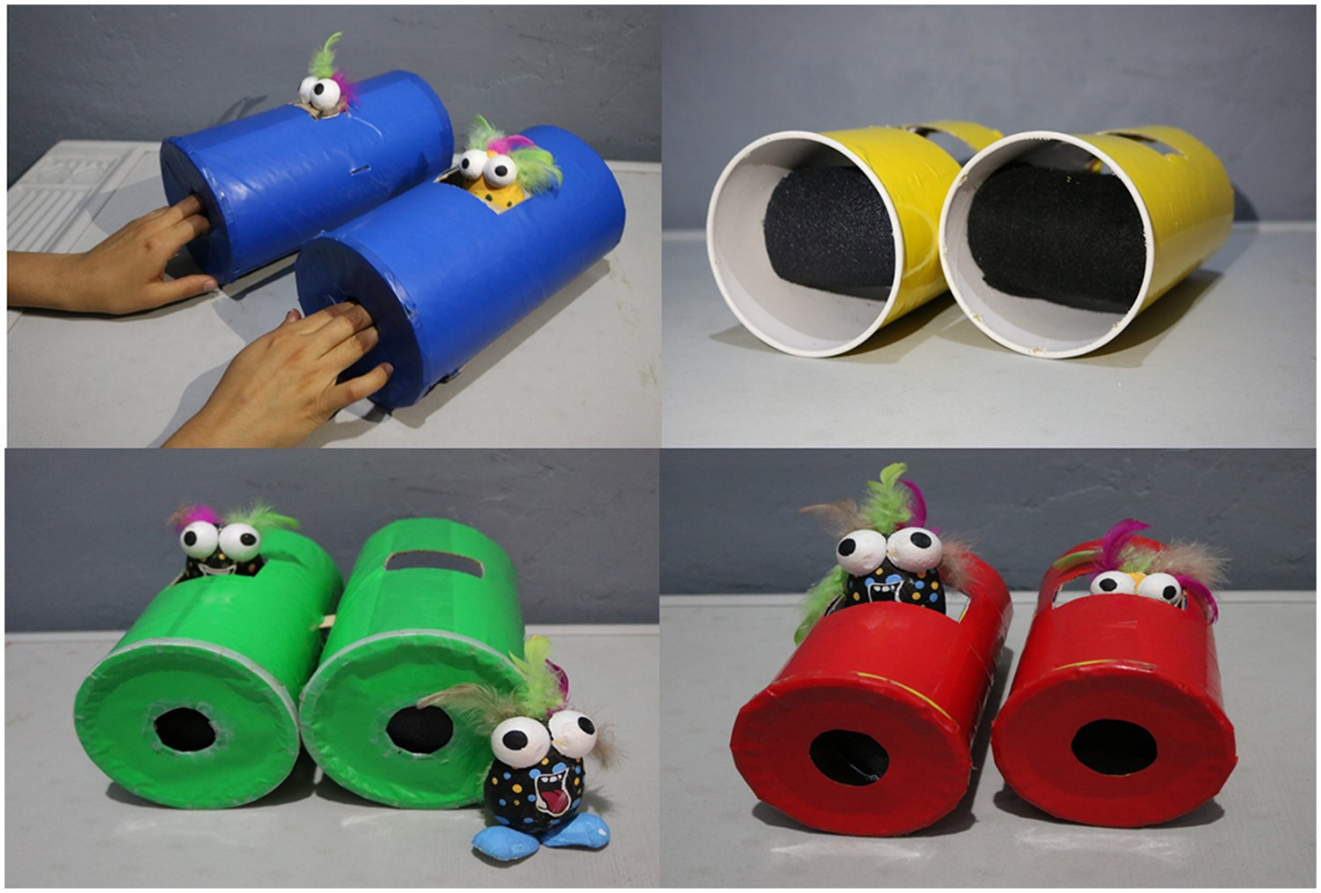
Materials for validation trials. Stuffed puppet that interacted with the children (top left). Sample toys with stimuli on their feet (top right). Toys and props that supported the story (bottom).

Before the first validation trial the experimenter narrated a story about a zoo whose animals had either *rough* or *smooth* feet. In telling the story, the child was introduced to the eight plastic toys (Fig 3, bottom): four with rough feet and four with smooth feet (Fig 3, top right).

To tell the story, a stuffed puppet called *Mr*. *Alce Matute* was introduced (Fig 3, top left). The experimenter said: ‘Hi, my name is Alce Matute! I can’t move that well, as my arm is hurting. Some animals have escaped from the zoo, and I must get them back into their pens. Would you help me find them and get them back? Would you? Very Good!’ (Original Spanish Script: *‘¡Hola amiguito*! *Mi nombre es Sr*. *Alce Matute*, *no puedo moverme muy bien porque mi brazo está lastimado*. *Se han escapado unos animales y debo meterlos en sus jaulas*. *¿Tú quieres ayudarme a encontrarlos*? *¿Sí*? *¡Muy bien*!*’*).

In the validation trials the experimenter (by means of the stuffed puppet) said to the child: ‘Let see, I have a friend that’s a lion with rough feet. Can you bring the rough-feet lion to me?’ This assessed that the child understood the question, and that he recognized the textures. The toys were presented in a counter-balanced manner. (Original Spanish Script: ‘*Vamos a ver*, *tengo un amigo que es un león con pies rasposos ¿puedes pasarme al león con pies rasposos*?’).

If the child picked the correct animal, the experimenter said, ‘Very Good. I’m happy you found my friend!’. If the child picked the incorrect animal, the experimenter would say, ‘Are you trying to trick me? You’re funny! Try again. Can you bring the rough-feet lion to me?’ Scripts along similar lines were used for the remaining validation trials. (Original Spanish Script; for correct answer: ‘*¡Yuju*! *Estoy feliz de que hayas encontrado a mi amigo león*’. For incorrect answer: ‘¿*Estás tratando de engañarme*? *Qué gracioso eres intenta otra vez*, *pásame al león con pies rasposos*’).

The procedure was repeated until four validation trials were conducted in the previously mentioned order (VT1-TT1-VT2-TT2-VT3-VT4-TT3-TT4), each time asking for a different (either with smooth or rough feet) plastic animal.

Four test trials were conducted. Before the first test trial, two cylinders containing tactile stimuli (as described in the *Stimuli and materials* section) were presented while telling a new part of the story: ‘Mr. Matute has given us another mission: we must find some strange creatures. They have funny names. One is called */krexis/* and the other is called */nunum/*’ (or */xikres/* and */munmu/* for the third and fourth trials). ‘We cannot see them, but we can touch them. You can put your hands inside (referring to the cylinders) and touch them. Go ahead, touch them carefully’. After the child touched the textures inside the cylinder, the experimenter said ‘Very good’. Next, she asked ‘Do you remember what are they called?’ She waited for the answer, in case the child did not respond the experimenter repeated the names (*/krexis/* and */nunum/* or */xikres/* and */munmu/*) and asked the child to repeat the pair of names. After this, the experimenter said ‘Yes, all right, one is called */krexis/*, the other */nunum/*’. The order of the nonwords tested was counterbalanced. (Original Spanish Script: -*‘Matute nos ha dado otra misión*: *Encontrar a unas criaturas extrañas de nombres chistosos*, *yo solo sé que uno se llama “krejis” y el otro se llama “nunun”*. *No podemos verlos*, *pero sí tocarlos*. *Puedes meter tus manos*, *tocarlos y sentirlos*, *¿ya los tocaste*? *-“¡Muy bien*! *… ¿Recuerdas cómo se llaman*?*’–‘Así es*, *uno se llama “nunum”*, *y otro se llama “krejis”*.*’*).

#### Response phase

In the response phase the experimenter asked ‘Can you tell me where is */krexis/*?’ (or any of the other three nonwords). The child responded taking his hand out of the cylinder and pointing to one of the cylinders. The pair of cylinders in the first and second trials were different in color from the third and fourth trials. The textures were placed in front of the child (left and right) in a counterbalanced order. (Original Spanish script: ‘*¿Cuál te parece que es krejis*?*’*).

## Results

**Fig 4:**
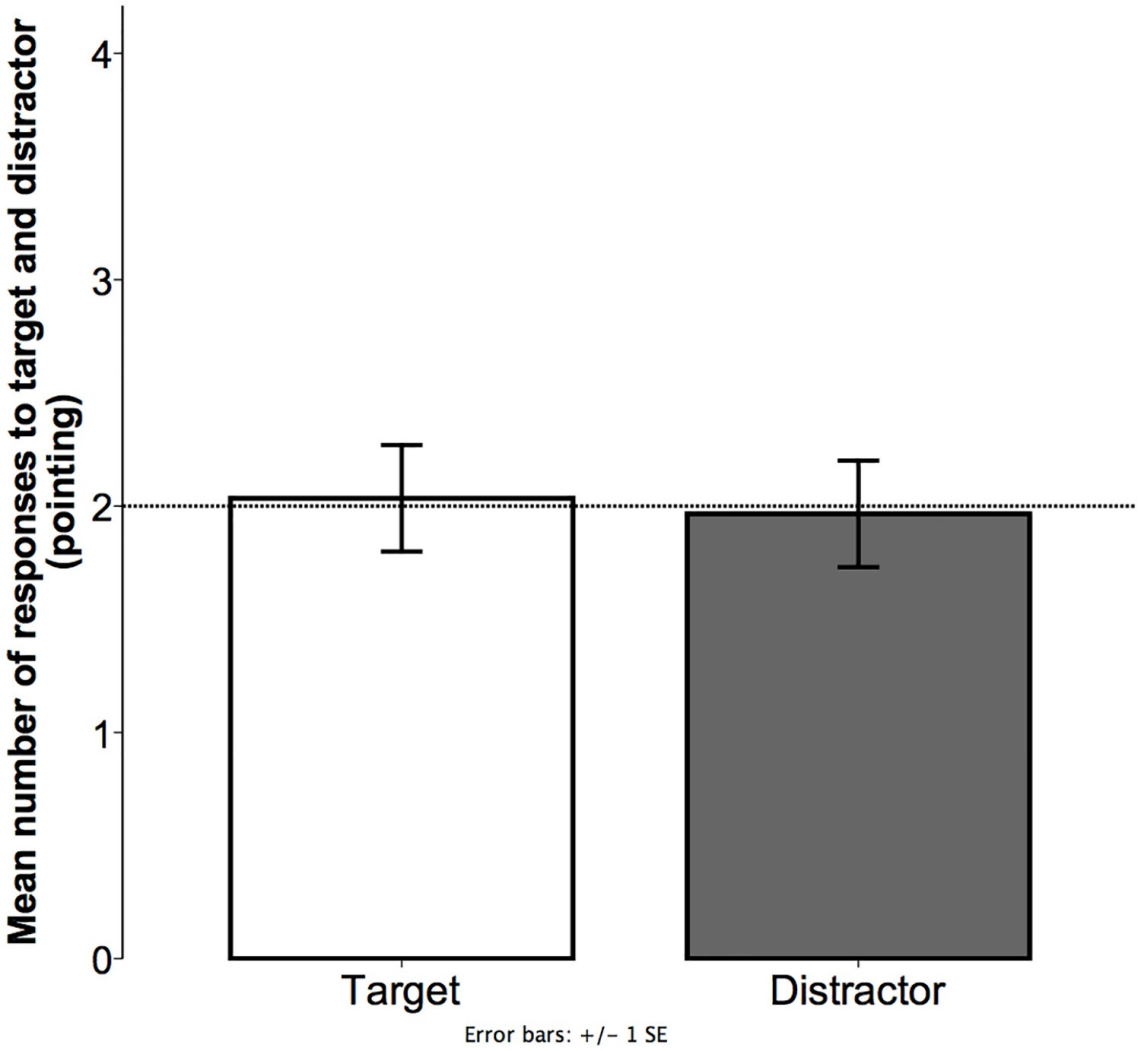
Experiment 1. Mean number of associations to target and distractor (pointing). Values were calculated based on the total number of trials per child (4). Bars show the mean number of trials children pointed to the target or the distractor texture. Dotted line depicts chance level. Error bars represent standard error of means.

**Fig 5:**
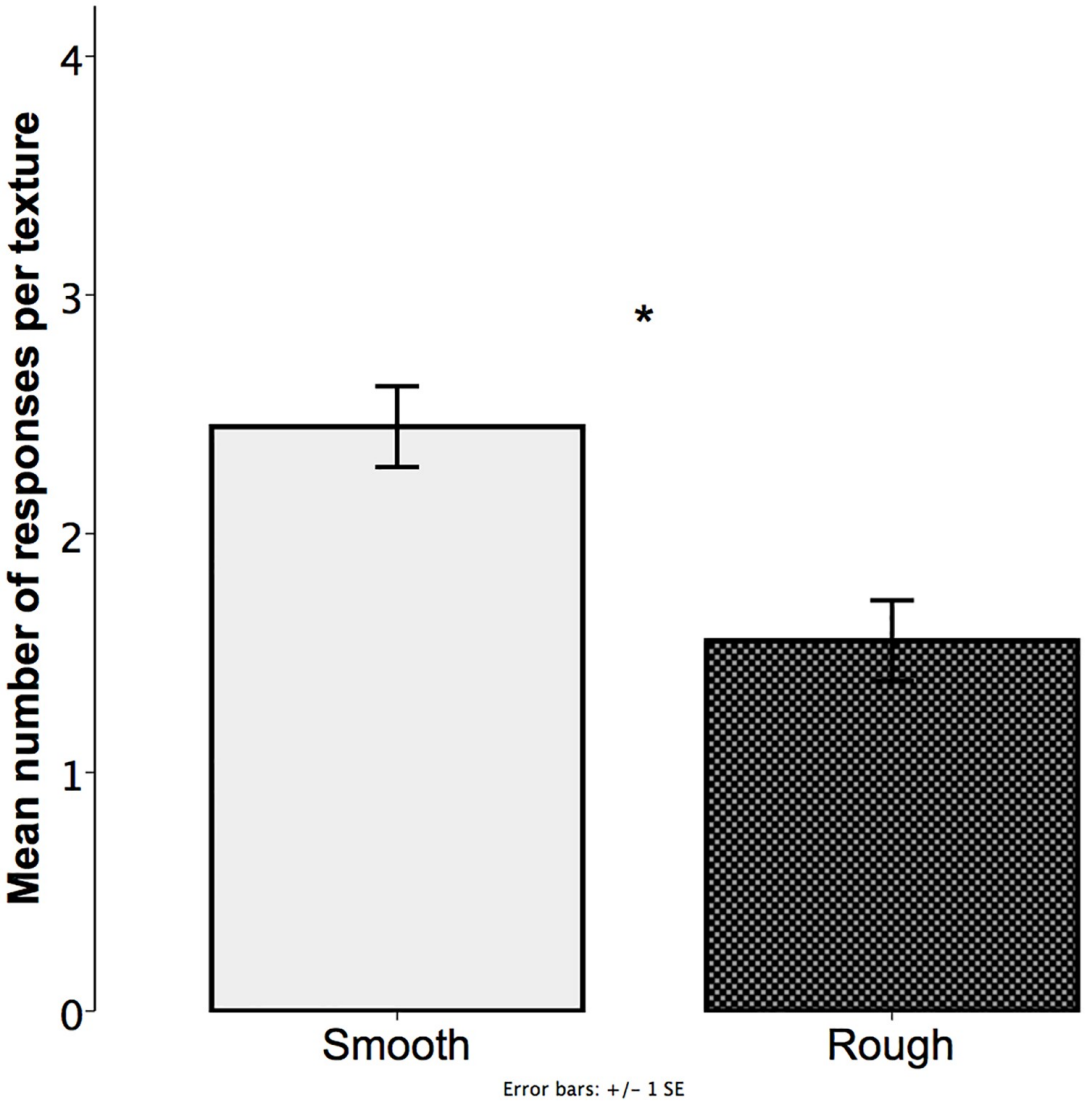
Experiment 1. Mean number of responses for each of the two textures. Values were calculated based on the total number of trials per child (4). Error bars represent standard error of means. *p < .05.

Responses were coded as follows: for each of the four trials, children were given a score of 1 when they responded as predicted by pointing to the target texture (i.e., pointing to the predicted texture; the rough stimulus corresponded to */krexis/* or */xikres/* and the smooth stimulus corresponded to */nunum/* or */munmu/*) according to the experimenter’s prompt (i.e., one of the two nonwords) and a score of 0 when they pointed to the distractor stimulus (see Fig 4). Experimenter also coded whether children chose the rough or the smooth texture (see Fig 5).

As the dependent variable was binary—target or distractor—we analyzed the responses using a mixed-effects logit model. The analysis showed no significant difference between children’s associations that were congruent with the prediction (*M* = 2.03; *SD* = 1.26) and chance (likelihood ratio = .036, χ2 = .036, df = 1, p = 0.849).

Additionally, similar analyses were conducted to determine whether children favored any of the textures. Regardless of the nonword pronounced by the experimenter, children showed a preference for the smooth stimulus (*M* = 2.44; *SD* = .90) compared with the rough stimulus (*M* = 1.55; *SD* = .90). This difference was statistically significant as shown in the model (likelihood ratio = 5.879, χ2 = 5.829, df = 1, p < 0.05).

In sum, no sound symbolic effect was observed in Experiment 1. However we observed a preference for the smooth stimulus. It is thus possible that the observed bias may be obscuring a latent sound symbolic effect. Experiment 2 may help us evaluate this possibility, since texture is eliminated as a choice in Experiment 2; instead, children assign nonwords to a given texture.

### Experiment 2. Naming task

#### Participants

Thirty children whose mean age was 4 years and 1 month (*SD* = 4 months; range = 3.5–4.5 years) participated in Experiment 2. Children were students from the same schools as in Experiment 1, and were included based on the same criteria. Experiment 2 was conducted on April 2018.

#### Procedure

Stimuli and materials were the same as the ones in Experiment 1. Procedure in Experiment 2 was also the same as in Experiment 1, except for the response phase. Children were asked to name the indicated texture (i.e., rough or smooth) by uttering one of the alternative nonwords. In the response phase the experimenter said, referring to the strange creatures inside the cylinders, ‘I don’t know which one is who, but you do, since you have already touched them’. The experimenter pointed to the cylinder on the left side, and asked ‘Can you tell me who this is?’ The child gave a verbal response, after the child responded, the experimenter said ‘Very good’. Next, she pointed to the cylinder on the right and asked ‘And who is this?’ This was done in order to make sure the child utilized and remembered both nonwords and that he was not simply picking one label (due to familiarity or bias.). (Original Spanish Script: *‘No sé cuál es cuál*, *pero tú sí*, *pues ya los tocaste*. Pointing to left cylinder: *-‘¿Me puedes decir quién es este*?*’–‘¡Muy Bien*!*’*. Pointing to right cylinder: *-‘¿Y quién es este*?*’*).

## Results

**Fig 6:**
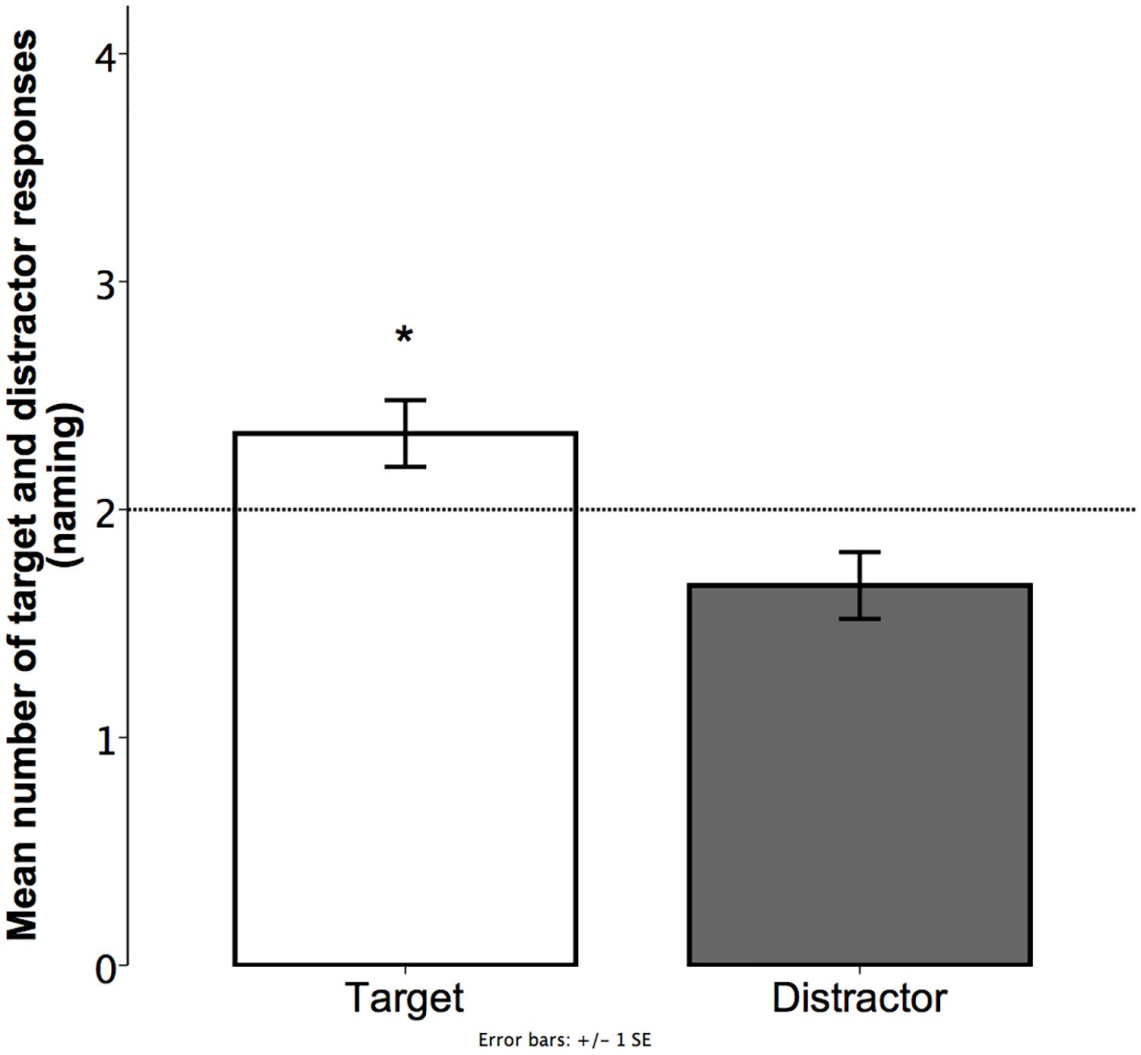
Experiment 2. Mean number of associations to target and distractor (naming). Values were calculated based on the total number of trials per child (4). Bars show the mean number of trials children named the target or the distractor nonword. Dotted line depicts chance level. Error bars represent standard error of means. *Children’s naming of the target nonword was significantly above chance (p < .05).

**Fig 7:**
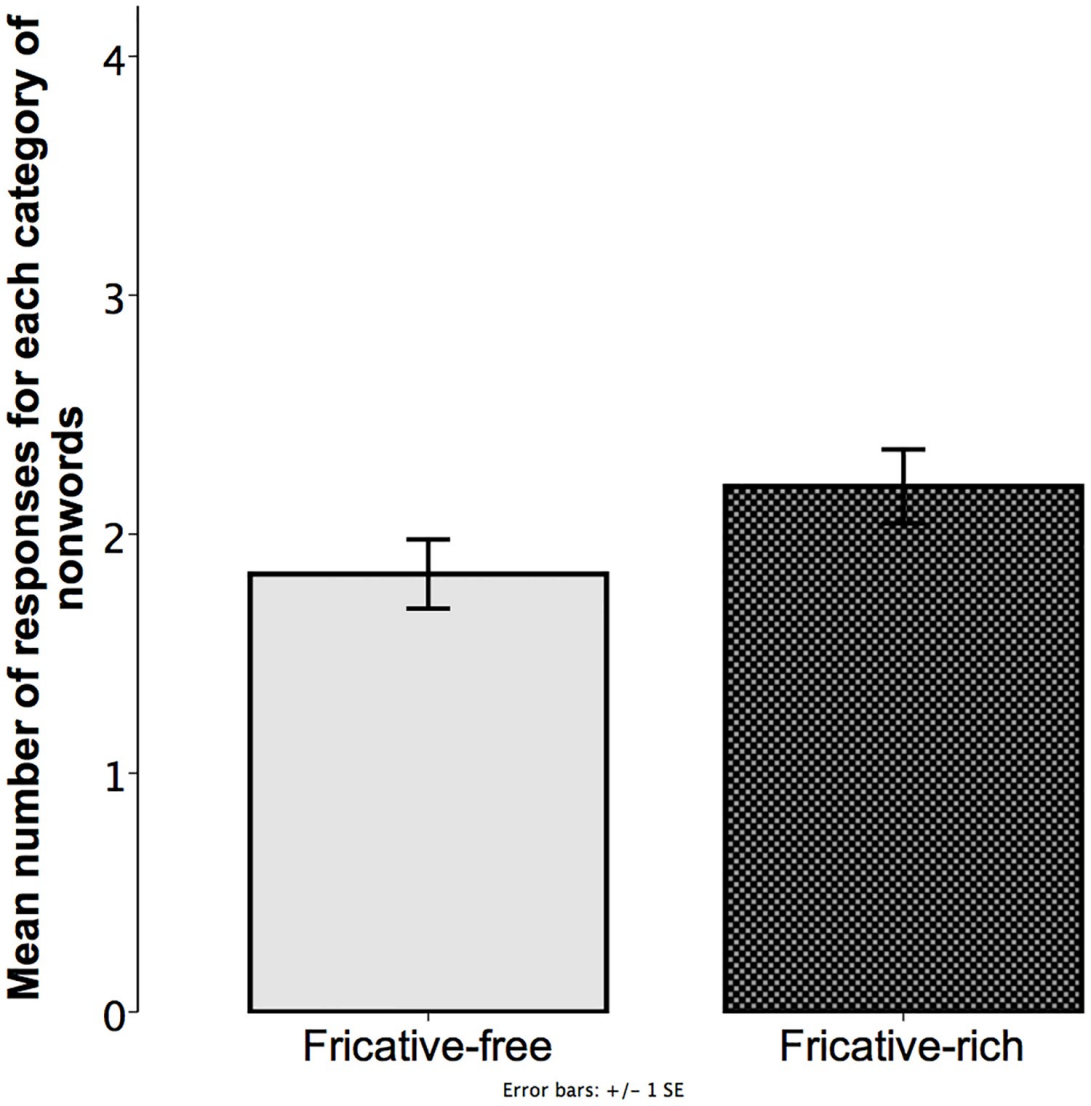
Experiment 2. Mean number of responses for each category of nonwords. Values were calculated based on the total number of trials per child (4). Bars show the mean number of trials children named a fricative-rich or a fricative-free nonword. Error bars represent standard error of means.

Children were given a score of 1 when they responded as predicted, by uttering the target word corresponding to texture the experimenter indicated *(/krexis/* or */xikres/* correspond to the rough stimulus and */nunum/* or */munmu/* correspond to the smooth stimulus), and a score of 0 when children uttered the distractor word (see Fig 6). Experimenter also coded whether children chose to name either a fricative-rich or a fricative-free nonword (see Fig 7).

Responses for Experiment 2 were analyzed using a mixed-effects logit model. Children’s performance was above chance (*M* = 2.33; *SD* = .80) as shown in the model (likelihood ratio = 4.085, χ2 = 4.062, df = 1, p < 0.05).

Children in Experiment 2 showed a sound symbolic effect, by systematically associating */krexis/* or */xikres/* to the rough stimulus (sandpaper) and */nunum/* or */munmu/* to the smooth stimulus (polar-fleece fabric).

Since we found a preference for the smooth stimulus in Experiment 1, we analyzed the data to determine whether there is an analogous preference for a type of word. When comparing the rate of naming either a fricative-rich nonword (*M* = 2.2; *SD* = .84) or a fricative-free nonword (*M* = 1.83; *SD* = .79), no significant effect was observed (likelihood ratio = .549, χ2 = .549, df = 1, p = 0.459).

## Discussion

Experiment 2 showed haptic sound symbolism in an early developmental stage. Most evidences of tactile crossmodal correspondences come from studying adults. Addressing tactile sound symbolism in young children adds a developmental aspect to the understanding of the phenomenon. Additionally, we implemented a dual-task experimental design in order to probe the effect of task difficulty and to control for biases in the tactile or auditory modalities. This design allowed us to explore how relative is the manifestation of the effect.

As mentioned, no sound symbolic effect was observed in Experiment 1. This is puzzling since Experiment 1 involved a, supposedly, less demanding pointing task, compared to the naming task in Experiment 2. However, the effect was found in the more demanding task.

An explanation for the bias observed in Experiment 1 (i.e., children’s bias for the smooth stimulus), may be that the preference is a hedonic response to touching the smooth stimulus; Etzi et al.’s [40] results support this view. Etzi et al. confirmed the existence of tactile-emotional and tactile-hedonic associations. In particular, rough stimuli showed correspondences with negative emotions and negative evaluations. Smooth stimuli showed correspondences with positive emotions and hedonic evaluations. Thus, the simplest explanation is that children experienced a hedonic response in touching the smooth stimulus. Hence the resulting preference for the smooth stimulus may be stronger than the latent sound symbolic effect. By contrast, in Experiment 2, children are asked for a verbal response, children do not exhibit a preference for one word or another. This avoids the smooth preference, allowing the sound symbolic effect to manifest itself. This possibility, however, comes with a caveat, since no difference in accuracy between the two experiments was observed.

Now, an alternative explanation for the sound symbolic effect in Experiment 2, is that the results are associated to the more active engagement demanded by the task. Support for this suggestion can be found in Oda [44]; who found that the sound symbolic effect was stronger when pronouncing Japanese ideophones, compared to only hearing them. This shows that articulation can modulate the sound symbolic effect. It must be pointed out that the child pronounces the nonwords in the pointing task as well, when the experimenter makes sure that the child knows and can pronounce the nonwords. However, an important difference is that pronouncing the words is not part of the pointing task itself (i.e., words were pronounced by the children only as a rehearsal and not as an answer to a requested association between the nonword and the texture).

In further support, we can cite the views that provide sound symbolism with a role in facilitating language acquisition [4,6,8,22,45]. In particular, Ohala [9,10,46] suggests that sound symbolism has a natural basis on the physiological constraints of the subjects. Ohala [9] argues that some characteristics of sound symbolism can be explained as the result of motor and other physiological constraints in the human vocal tract. Moreover, Ohala identifies sound symbolism as an instance of a system for conveying meaning that exploits natural correspondences [10,47]. For example, large animals produce low-pitched sounds. A large animal is also menacing. Thus, animals incorporate the *low-pitched = large* correspondences into their behaviors and tend to respond to low-pitched sounds as indicating *large* and *menacing*. Now, Ohala’s view supports the suggestion that articulation modulates the sound symbolic effect, since articulating words is a circumstance that better replicates the constraints that children encounter in their everyday usage of language, contrasting with the silent situation in the pointing task. The naming task may better match the natural settings in which sound symbolism originates. This matching may be the factor liable for the fact that articulation intensifies the sound symbolic effect. This possibility has to do with a general hypothesis that sound symbolic-effects may result from indirect psychological associations. More specifically, the hypothesis is that sound, on the one hand, and shape, on the other, may systematically induce a similar indirect experience in the subject. That is, the word *kiki*, for instance, may systematically evoke the same thought or feeling as the perception of a spiky shape. It is in recognizing the similarity of these *thoughts* (not the *stimuli*) where the sound-symbolic effect emerges. Instances of this hypothesis have been suggested by Masuda [48] and von Humboldt [49]. More specific to this study, the friction in fricative consonants may be analogous to the friction in touching materials, and it may be in recognizing the analogy where the effect emerges. However, this remains an open question to be tested empirically.

Finally, we must point out the possibility that the observed effects may result from associations or biases related to the specific stimuli utilized in the study. One limitation of our experimental design is that the smooth-rough dimension was probed using only two types of materials. Studies such as Etzi et al. [40] or Sakamoto and Watanabe’s [38] tested a wider variation of materials. The rationale underlying the decision of utilizing fewer materials was to rely on prior knowledge of word-tactile correspondences, in order to simplify the task. Further studies may confirm our results using different materials.

## Conclusions

We have reported evidence for tactile sound symbolism in Spanish-speaking young children. In testing rough (sandpaper) and smooth (polar-fleece fabric) tactile stimuli, a systematic association between fricative-rich nonwords and the rough stimulus, and between fricative-free nonwords and the smooth stimulus was found when children were prompted to give a verbal response. To the best of our knowledge, our results, although limited in scope, represent the first contribution regarding tactile-word correspondences in the 3.5–4.5 years old developmental stage.

In addition, an unexpected effect was observed: in Experiment 1, in which children had to select the stimulus without articulating the word, no sound symbolic effect was found. Rather, a bias towards selecting the smooth stimulus was observed. In Experiment 2 (the more demanding task), a sound-symbolic effect was observed. To explain these counter-intuitive results, we suggested that the preference in Experiment 1 may be the result of hedonic bias, and that these preferences are capable of obscuring the sound symbolic effect, once this preference was eliminated sound symbolic sensitivity was captured. We also suggested an alternative explanation: articulation may be a factor for sound symbolic associations, since pronouncing words may match the conditions that originate sound symbolism. To ascertain any of these hypotheses additional studies are necessary, which should further illuminate the development of tactile sound symbolism.

## Supporting information

S1 File Data-Experiments 1 and 2.zip. Contains coded responses regarding target and distractor and preferences for the type of response. All data is presented summarized by child.
(ZIP)
